# Supplementary material for: Neurodevelopmental disorder mutations in the purine biosynthetic enzyme IMPDH2 disrupt its allosteric regulation
Source: J Biol Chem. 2023 Jul 4;299(8):105012. doi: 10.1016/j.jbc.2023.105012 (PMC10407431; doi:10.1016/j.jbc.2023.105012)
Supplement: Supporting Information [file mmc1.docx]

Supporting Information for

**Neurodevelopmental disorder mutations in the purine biosynthetic enzyme IMPDH2 disrupt its allosteric regulation**

Audrey G O’Neill^1^, Anika L Burrell^1^, Michael Zech^2,3^, Orly Elpeleg^4,5^, Tamar Harel^4,5^, Simon Edvardson^6^, Hagar Mor Shaked^4,5^, Alyssa L Rippert^7^, Tomoki Nomakuchi^7^, Kosuke Izumi^7^, Justin M Kollman^1*^

^1^Department of Biochemistry, University of Washington, Seattle, WA, USA.

^2^Institute of Neurogenomics, Helmholtz Zentrum München, 85764 Munich, Germany.

^3^Institute of Human Genetics, School of Medicine, Technical University of Munich, 81675 Munich, Germany.

^4^Department of Genetics, Hadassah Medical Center, Jerusalem, Israel.

^5^Faculty of Medicine, Hebrew University of Jerusalem, Jerusalem, Israel.

^6^Alyn Hospital, Hebrew University School of Medicine, Jerusalem, Israel.

^7^Division of Human Genetics, Children’s Hospital of Philadelphia, Philadelphia, PA, USA.

*Correspondence should be addressed to Justin Kollman; Email: jkoll@uw.edu

**This PDF file includes:**

Clinical Reports

Tables S1 to S3

Figs. S1 to S8

**Clinical Reports**

**Clinical report for case subject with L245P variant of IMPDH2**

The proband was a 3-year-old female, the firstborn and only child of non-consanguineous parents of Jewish Ashkenazi origin. Pregnancy was significant for intrauterine growth retardation (IUGR), dilation of the lateral ventricles, and mild pulmonic stenosis on fetal ultrasound. Delivery was at 33+6 weeks, at a birthweight of 1520 grams (7th %ile) and with a head circumference of 28.5 cm (9th %ile). Complications of prematurity included bronchopulmonary dysplasia (BPD) with oxygen dependency for approximately one month. She was born with congenital dysplasia of the hip (CDH) which required surgical intervention, and with left sided torticollis. Echocardiogram revealed pulmonic stenosis, for which she underwent interventional catheterization (balloon angioplasty). There was also an atrial septal defect which closed spontaneously. Head ultrasound showed mild dilation of the left ventricle. Renal ultrasound was normal.

The proband had significant hypotonia and global developmental delay. She walked at around 2-years-7-months of age, and at 3 years of age, said less than 10 words, although she used nonverbal communication and seemed to have a higher receptive ability. Development quotient (DQ) was 42 at 14 months, and 60 at repeat evaluation. Hearing evaluation showed bilateral type B tympanograms and normal free field audiometry. She had abnormal posturing of the head, which she held tilted back and had a downward gaze. Ophthalmology exam including ultrasound of the orbits was normal. There were no convulsive episodes, and EEG was normal.

Family history was positive for CDH in the proband's father and the paternal uncle. The mother's half-brother was born with omphalocele and died at 2 years of age. His cognition was reported to be normal, and no DNA was available for testing. The maternal grandfather died in his 20's of cancer affecting his leg; no further information was available.

On physical examination at 3-years-2-months, the child had failure to thrive, with a preserved head circumference of 47.5 cm (~10th %ile). She was responsive, had good eye contact yet minimal facial mimicry. She held her head tilted back with abnormal posturing. She had a double hair whorl, sparse thin hair, high forehead, hypertelorism, bilateral epicanthal folds, a broad nasal bridge, open mouth, small low-set and posteriorly rotate ears, a systolic murmur, and axial hypotonia.

**Clinical report for case subject with K238R variant of IMPDH2**

The proband was the product of a naturally conceived single gestation to a then 27-year-old G2P2 mother. Pregnancy and neonatal history were uncomplicated. Family history was non-contributory. He was born at 40+1 weeks gestation weighing 2920g (25th percentile) and was 51.4cm in length (75th-90th percentile). HC at birth was 33cm (25th percentile). APGAR scores were 8 at 1 minute and 9 at 5 minutes. He was discharged home at day of life 2.

Concerns for his development were first raised at 6.5 months of age due to gross motor delays. He sat at 8-9 months, crawled at 12 months, and walked at 18 months. Significant speech delay was also noted. Medical history was notable for tachypnea and stridor with feeds as a neonate, milk protein allergy and gastroesophageal reflux, meatal stenosis and penile adhesion s/p correction, easy bruising, torticollis and plagiocephaly, eczema, and hypotonia.

At 9 months of age, he weighed 8.396kg (24th percentile), was 74.8cm in length (80th percentile), and head circumference was 44.1cm (18 percentile). Physical examination was notable for slight upslant to palpebral fissures, prominent cheeks, supernumerary nipple, and mild hypotonia.

Follow-up at 23 months was notable for persistent developmental delay with most skills clustering around the 16-20 month age range per Developmental Pediatrician and concern for possible autism spectrum disorder. He was referred to Neurology for evaluation of possible movement disorders and was again noted to have hypotonia. He was started on Sinemet (6mg BID) due to the possibility of L-DOPA responsive symptoms in IMPDH2 related disorders with reported improvements in energy, interaction, speech skills, and movement.

| **Variant** | **IMP** | | **NAD^+^** | | **V_max app_** |
| --- | --- | --- | --- | --- | --- |
|  | **K_0.5_ (µM)** | **Hill** | **K_0.5_ (µM)** | **Hill** | **(µM NADH min^-1^)** |
| **WT** | 45.6 | 7.4 | 44.9 | 5.3 | 2.2 ± 0.1 |
| **G113E** | 17.6 | 2.3 | 44.9 | 2.8 | 2.4 ± 0.8 |
| **G113R** | 20.1 | 2.8 | 31.5 | 3.3 | 2.0 ± 0.5 |
| **G207R** | 35.5 | 2.8 | 37.1 | 5.8 | 2.1 ± 0.1 |
| **S160del** | 15.1 | 2.8 | 25.8 | 5.7 | 1.7 ± 0.2 |
| **Q243H** | 52.1 | 4.9 | 60.2 | 3.0 | 3.6 ± 0.5 |
| **L245P** | 16.9 | 1.7 | 24.6 | 3.5 | 2.8 ± 0.4 |
| **K238R** | 17.2 | 2.5 | 33.4 | 9.0 | 1.4 ± 0.1 |

**Table S1. Kinetic parameters of IMPDH2 variants compared to WT IMPDH2.**

| **Variant** | **ATP** | **GTP** |
| --- | --- | --- |
| WT | Extended filaments | Compressed filaments |
| G113E | Compressed filaments | Compressed filaments |
| G113R | Compressed filaments | Compressed filaments |
| G207R | Extended filaments | Compressed filaments |
| S160del | No filaments | Octamers |
| Q243H | Extended filaments | Compressed filaments |
| L245P | Extended filaments | Compressed filaments |
| K238R | Extended filaments | Compressed filaments |

**Table S2. Summary of negative stain results from Fig. 4.**

|  | **hIMPDH2-L245P extended filament interface** | **hIMPDH2-L245P extended filament segment** | **hIMPDH2-L245P compressed filament interface** | **hIMPDH2-L245P compressed filament segment** | **hIMPDH2-L245P bent filament segment** |
| --- | --- | --- | --- | --- | --- |
| **Ligands** | ATP, IMP, NAD+ | ATP, IMP, NAD+ | GTP, ATP, IMP, NAD+ | GTP, ATP, IMP, NAD+ | GTP, ATP, IMP, NAD+ |
| **PDB ID** | 8FOZ | 8G8F | 8FUZ | 8G9B | N/A |
| **EMDB ID** | EMD-29357 | EMD-29848 | EMD-29482 | EMD-29863 | EMD-29870 |
| **Data collection and refinement** | | | | | |
| **Magnification** | 105,000 | 105,000 | 105,000 | 105,000 | 105,000 |
| **Voltage (kV)** | 300 | 300 | 300 | 300 | 300 |
| **Electron exposure (e^-^/Å^2^)** | 60 | 60 | 60 | 60 | 60 |
| **Defocus range (µm)** | -1.71 — -0.27 | -1.71 — -0.27 | -1.74 — -0.28 | -1.74 — -0.28 | -1.74 — -0.28 |
| **Pixel size (data collection) (Å)** | 0.4215 | 0.4215 | 0.4215 | 0.4215 | 0.4215 |
| **Pixel size (reconstruction) (Å)** | 0.843 | 0.843 | 0.843 | 0.843 | 0.843 |
| **Micrographs (no.)** | 2,648 | 2,648 | 2,967 | 2,967 | 2,967 |
| **Initial particles (no.)** | 1,893,802 | 1,893,802 | 1,421,990 | 1,421,990 | 1,421,990 |
| **Final particles (no.)** | 152,170 | 54,304 | 89,981 | 26,540 | 91,716 |
| **Symmetry imposed** | D4 | D4 | D4 | D4 | C1 |
| **Map resolution range,**  **Relion postprocess (Å)** | 2.06 – 3.37 | 2.63 – 4.59 | 2.13 – 4.10 | 3.05 – 5.21 | 2.76 – 5.91 |
| **Resolution, Relion postprocess**  **(0.143 FSC) (Å)** | 2.1 | 2.8 | 2.2 | 3.2 | 3.0 |
| **Resolution, density modified**  **(0.5 FSC_ref_) (Å)** | 2.0 | 2.6 | 2.1 | 3.0 | 2.7 |
| **Model refinement and validation** | | | | | |
| **Initial model (PDB ID)** | 6U8E | 6U8N | 6U8S | 6U9O | N/A |
| **R.m.s. deviations** |  |  |  |  |  |
| **Bond lengths (Å)** | 0.0083 | 0.0136 | 0.0109 | 0.0164 | N/A |
| **Bond angles (°)** | 0.80 | 1.25 | 1.01 | 1.27 | N/A |
| **MolProbity score** | 1.17 | 2.05 | 1.31 | 1.84 | N/A |
| **Clashscore** | 2.94 | 7.63 | 4.61 | 10.84 | N/A |
| **C-beta deviations** | 0 | 0 | 0 | 1 | N/A |
| **Rotamer outliers (%)** | 1.31% | 1.87% | 1.17% | 0.60 | N/A |
| **Ramachandran plot** |  |  |  |  |  |
| **Favored (%)** | 99.47% | 93.61% | 97.92% | 95.85% | N/A |
| **Allowed (%)** | 0.53% | 5.98% | 2.08% | 3.99% | N/A |
| **Disallowed (%)** | 0.00% | 0.41% | 0.00% | 0.15% | N/A |

**Table S3. Cryo-EM data collection, refinement, and validation.**

**Figure S1. Photos of patient with the K238R variant at different ages.** Photos of patient at 9 months (A), 16 months (B), and 26 months of age (C).

**Figure S2. The L245P mutant is sensitive to high concentrations of GTP.** GTP inhibition curve of the L245P variant up to 16 mM GTP. Each data point represents the average initial rate of three reactions. Error bars represent standard deviation for n=3 technical replicates. Velocities were calculated from the change in absorbance at 340 nm. Reactions were initiated with 300 µM NAD+ and contained 1 µM enzyme, 1 mM ATP, 1 mM IMP, 1 mM MgCl2 and varying concentrations of GTP.

**Figure S3. Non-assembly disease mutants imaged using negative stain EM.** Representative negative stain images of 0.5 µM enzyme with either 1 mM ATP and 1 mM MgCl_2_ (A) or 5 mM GTP (B). Filaments were not observed in either condition.

**Figure S4. Extended L245P filament cryo-EM data processing.**

**Figure S5. Volume around ligands in extended and compressed L245P structures.** All ligands are resolved in the L245P extended and compressed structures. The catalytic domain is colored in green, and the regulatory domain is colored in pink.

**Figure S6. L245P+GTP/ATP/IMP/NAD+ data processing.**

**Figure S7. FSC curves of cryo-EM reconstructions.**

**Figure S8. Filament assembly interface reconstruction of the L245P filament in the presence of 20 mM GTP, 1 mM ATP, 3 mM IMP, 5 mM NAD+, and 1 mM MgCl2.**
